# Enabling image optimisation and artificial intelligence technologies for better Internet of Things framework to predict COVID

**DOI:** 10.1049/ntw2.12052

**Published:** 2022-08-31

**Authors:** Noor M Allayla, Farah Nazar Ibraheem, Refed Adnan Jaleel

**Affiliations:** ^1^ Department of Computer Engineering University of Mosul Mosul Iraq; ^2^ Department of Information and Communication Engineering Al‐Nahrain University Baghdad Iraq

**Keywords:** COVID, deep learning, image processing, Internet of Things, Support Vector Machine, Whale Optimisation Algorithm

## Abstract

Sensor technology advancements have provided a viable solution to fight COVID and to develop healthcare systems based on Internet of Things (IoTs). In this study, image processing and Artificial Intelligence (AI) are used to improve the IoT framework. Computed Tomography (CT) image‐based forecasting of COVID disease is among the important activities in medicine for measuring the severity of variability in the human body. In COVID CT images, the optimal gamma correction value was optimised using the Whale Optimisation Algorithm (WOA). During the search for the optimal solution, WOA was found to be a highly efficient algorithm, which has the characteristics of high precision and fast convergence. Whale Optimisation Algorithm is used to find best gamma correction value to present detailed information about a lung CT image, Also, in this study, analysis of important AI techniques has been done, such as Support Vector Machine (SVM) and Deep‐Learning (Deep‐Learning (DL)) for COVID disease forecasting in terms of amount of data training and computational power. Many experiments have been implemented to investigate the optimisation: SVM and DL with WOA and without WOA are compared by using confusion matrix parameters. From the results, we find that the DL model outperforms the SVM with WOA and without WOA.

## 1 INTRODUCTION

Internet of Things (IoTs) envisions billions of gadgets that can communicate, perceive and exchange data, it can then be examined to extract valuable information for management, decision‐making and planning. For a wide range of applications, the IoT holds great potential, such as manufacturing, healthcare, agriculture, transportation and telecommunication. Despite the IoT's popularity and the benefits it promises, it has been adopted much more slowly than expected. Some of the main reasons behind this are as follows: (1) policy, privacy, trust and security problems; (2) long capital cycles and lack of specialised labour are some of the challenges that must be overcome in order to use IoT properly; and (3) in some industries, there are not enough compelling use cases with a clear return on investment (ROI) [1, 2].

The effects of COVID have been so widespread that life as we know it may never be the same again. COVID has created or expanded digital technology applications and use cases, proving to be a catalyst for digital transformation [3]. Companies have had to adapt as a result, individuals and governments to adapt/change their views and priorities on ethical/societal problems [4]. Because IoT penetration has been slower than projected in several sectors, numerous of the problems have been caused, and in the result of this, these problems must resolved or minimized. IoT and other technologies, for example, have been used by governments to combat COVID [5]. COVID has caused lifestyle modifications such as study/work from home have also delivered new IoT use cases with demonstrable ROI, such as remote employee collaboration, workforce tracking and remote asset control. As a result, many businesses have boosted their investment in IoT and the speed at which they implement IoT projects. Because of the campaign against COVID‐19, attitudes towards privacy issues have softened, technology trust has risen and the approval process has been expedited [6]. As a result, IoT adoption in numerous industries is speeding up. The IoT uptake in smart buildings is also being accelerated by legal developments, such as more stringent cleaning and tracking requirements for enterprises [7].

Medical diagnosis and the development of novel pharmaceuticals have been greatly aided by Artificial Intelligence (AI). Artificial Intelligence is expected to have a major impact on radiologists by providing them with more accurate results with methods for accurate and faster diagnoses resulting in improved outcomes. Because computers will be able to process massive volumes of patient data, big data and AI will transform the way radiologists work, allowing them to specialise on a narrow range of duties [8]. Artificial Intelligence has already been successful in tackling problems, such as skin cancer and chronic diseases. Artificial Intelligence is expected to play a big role in the search for a cure for the corona virus in the near future, and as a result, individuals all across the world are experiencing less anxiety [9].

Medical imaging technology has advanced rapidly in recent years, with the advancement of computer technology, many picture processing and analysis methods are regularly updated. In addition, the usage of various image processing technologies in clinical practice is on the rise. As a result, the quality of computer graphics must be constantly increased in order to deliver more precise precision. Computer image processing technology development in medical imaging must be strengthened, continue to innovate in the field of image processing technologies, as a safety net for ensuring the future usability of medical clinical images [10]. It is one of the techniques used in image recognition and processing. Computer and artificial analysis of image and procedures of processing are more conducive to the enhancement of images. In today's world, augmentation of image is a form of image processing, and this is due to its advantages of ease of use and impressive results after processing [11]. Image processing and AI technologies will benefit greatly from this technology's advancements to improve the IoT framework. The aim of the proposed system is to test whale optimisation algorithm (WOA), Support vector machine (SVM) and deep‐learning (DL) algorithms in image processing and to offer how these technologies can be used to improve the IoTs. The contribution is that we are integrating the WOA with the image, SVM, DL and IoTs for COVID diagnosis.

The rest of this paper is arranged as follows: Section 2 presents the related works, Section 3 presents the algorithms and the proposed system, Section 4 presents the analysis of the outcomes for the suggested system and finally Section 5 offers the conclusions.

## 2 RELATED WORKS

Several papers published in the last year or so have explored the importance of the fight against COVID or future pandemics; IoT and other digital technologies are being used. The authors in Ref. [12] examine the many IoT‐based strategies employed to combat COVID. In Ref. [13], the authors examine the influence of the pandemic on various technologies as well as their social ramifications. In countries with a high rate of COVID cases, a comprehensive analysis of digital health options is offered in Ref. [14]. A consensus among Chinese specialists on the IoT‐assisted COVID diagnosis and therapy is reported in Ref. [15].

The costs, time and efficiency of implementing IoT in healthcare are listed in Ref. [16]. In this paper, the use of Big Data, IoT, AI and Block‐chain in reducing the impact of COVID are investigated in Ref. [17]. The authors in Ref. [18] consider whether IoT solutions can be beneficial in the fight against COVID. The authors in Ref. [19] discuss how various technologies of industry 4.0, such as IoT, AI and so on can aid in the prevention of disease spread. The use of AI in COVID has been suggested [20]. The COVID pandemic is examined in depth and the role of block‐chain, drones, IoT, AI and 5G in mitigating its effects have been offered in Ref. [21]. The authors in Ref. [22] contend that facilities should be in charge of contact tracing and recommend a contact‐tracking architecture, which is totally automated and does not depend on user participation.

The authors in Ref. [23] discuss three primary phases and numerous IoT healthcare applications: quarantine time, recovery and early diagnosis. Machine learning (ML), AI and other intelligent techniques to COVID prognosis were discussed in a recent survey [24]. The authors in Ref. [25] have collected a list of prospective IoT‐dependent COVID solutions. They provide a comprehensive review of the current state of IoT systems at variant layers, such as network, perception, cloud and fog layers. They also talk about how IoT can be used to diagnose COVID symptoms. Architecture has been suggested to assist the combat against COVID in [26], which contain four layer that depend on technologies of Block‐chain and IoT. They also talk about how IoT can be used to diagnose symptoms of COVID.

In addition, it incorporates a number of programmes designed to locate and track down COVID sufferers. The IoT has been considered in relation to the current digital healthcare infrastructure in Ref. [27]. It also discusses the impact of IoT‐enabled healthcare infrastructure on the actions of policymakers. In addition, existing enablers and impediments to IoT‐based healthcare adoption have been mentioned. In response to COVID, a comprehensive survey of IoT's impact on healthcare is offered in Ref. [28]. This is a comprehensive review of current advances in the Internet of Things in Healthcare (HIoT).

It also compares different IoT system implementation tactics prior to and throughout the epidemic. To combat COVID‐19, the authors in Ref. [29] compiled a list of early efforts to apply digital technologies in healthcare, considering variant classification, such as prevention, surveillance and diagnosis. The authors in Ref. [30] underline the critical need for AI to be used to battle future pandemics. These findings also show how AI‐based approaches to eradicating the epidemic are limited in scope. Finally, they conclude that science of data should be applied in global health to develop more accurate forecasting that can be applied by policymakers. The authors in Ref. [31] describe how the COVID pandemic might be combated using AI and the IoT.

Our paper differs greatly from the papers cited above in a number of ways; we take data as an image and according to image processing techniques and AI, the framework of IoT has been improved by using WOA algorithm to select best features from COVID images.

## 3 MATERIALS AND METHODS

**FIGURE 1:**
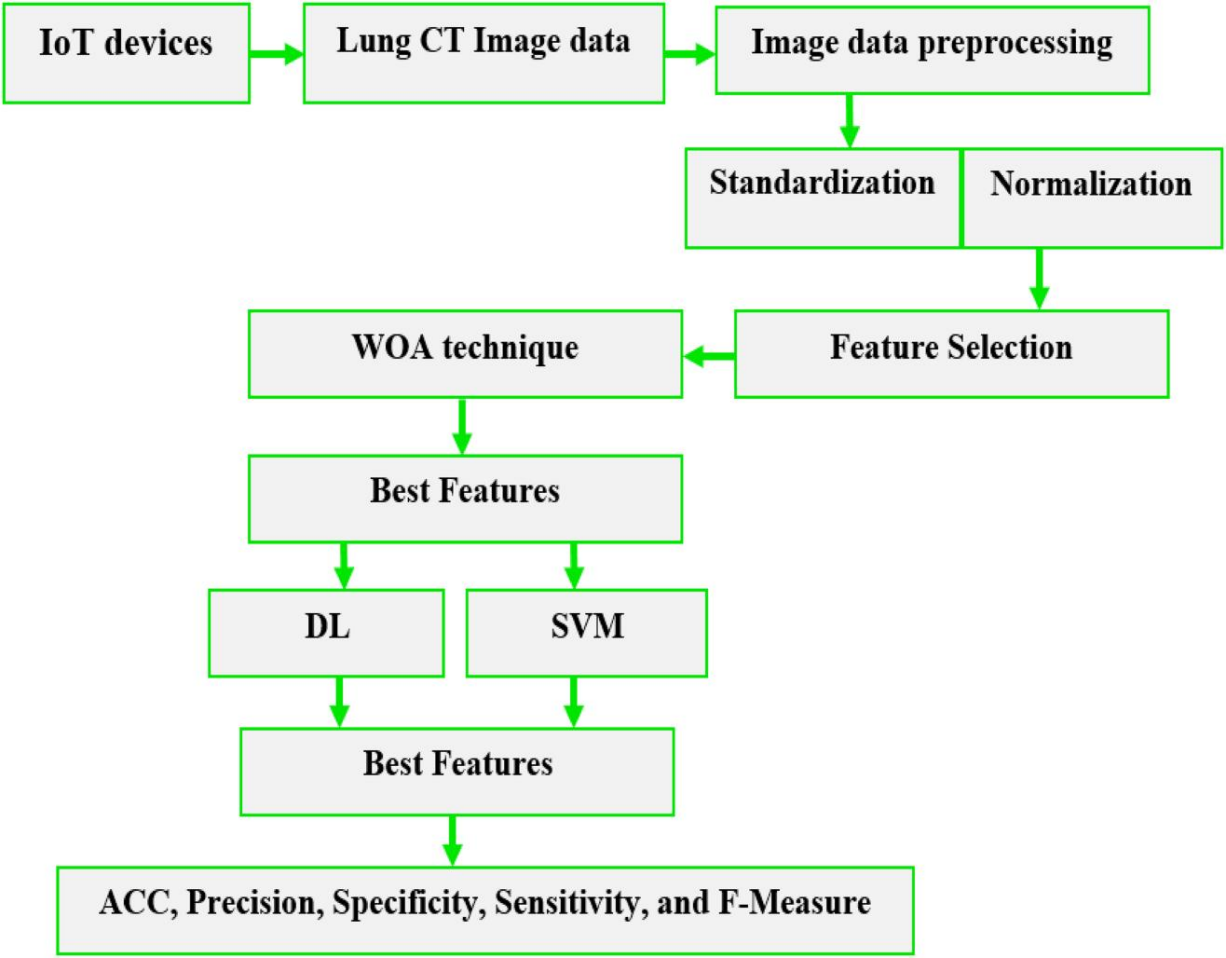
Framework of the proposed system

China's National Centre for Bio‐information (CNBI) provided the Computed Tomography (CT) lung imaging collection that we utilised in our investigation. These datasets were made accessible by the China National Centre to aid researchers in their fight against the COVID pandemic [32]. The novelty is in integrating the WOA with image, SVM, DL and IoTs. Figure 1 shows a block diagram of the proposed system. Medical sensors were used on the human body to collect features in real time, such as temperature, breath rate, headache and so on.

### 3.1 Image processing

**FIGURE 2:**
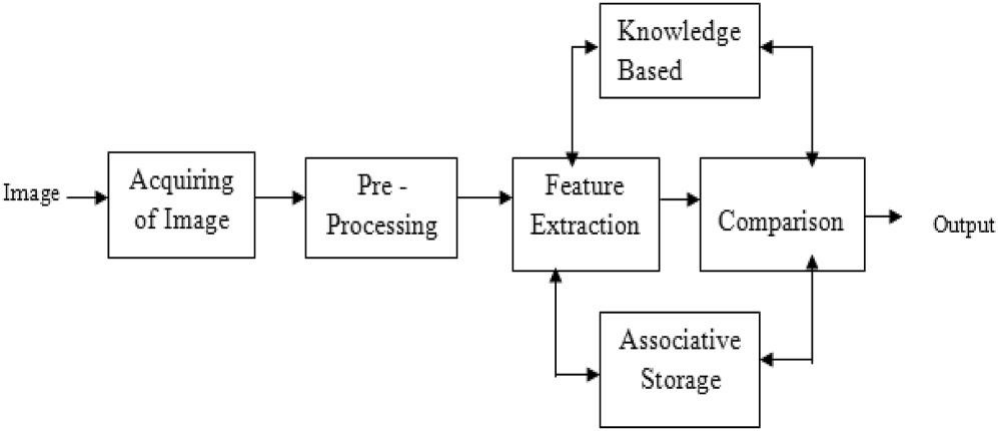
Image processing

Because data is often collected from a variety of sources, it is critical to have a plan in place to keep track of all the details. Image preprocessing reduces the complexity of data and improves the accuracy of the results. In order to supply the network with a clean dataset, this approach goes through multiple rounds of standardisation. Figure 2 offers the image processing block diagram. The following stages are used to do data preprocessing in the framework of this paper [33, 34]:

- Standardisation of Image: Unified aspect ratio images required for DL that deal with images. As a result, the first step is to reduce the size of the photos into a square shape and unique dimensions.
- Normalisation: To improve the training phase convergence of any algorithm of AI, input pixels must have a normalised distribution of data. It is defined as subtracting the distribution's mean and dividing by its standard deviation. Scaling normalised data is considered at the end of this stage to produce positive values.

### 3.2 Whale optimisation algorithm

**FIGURE 3:**
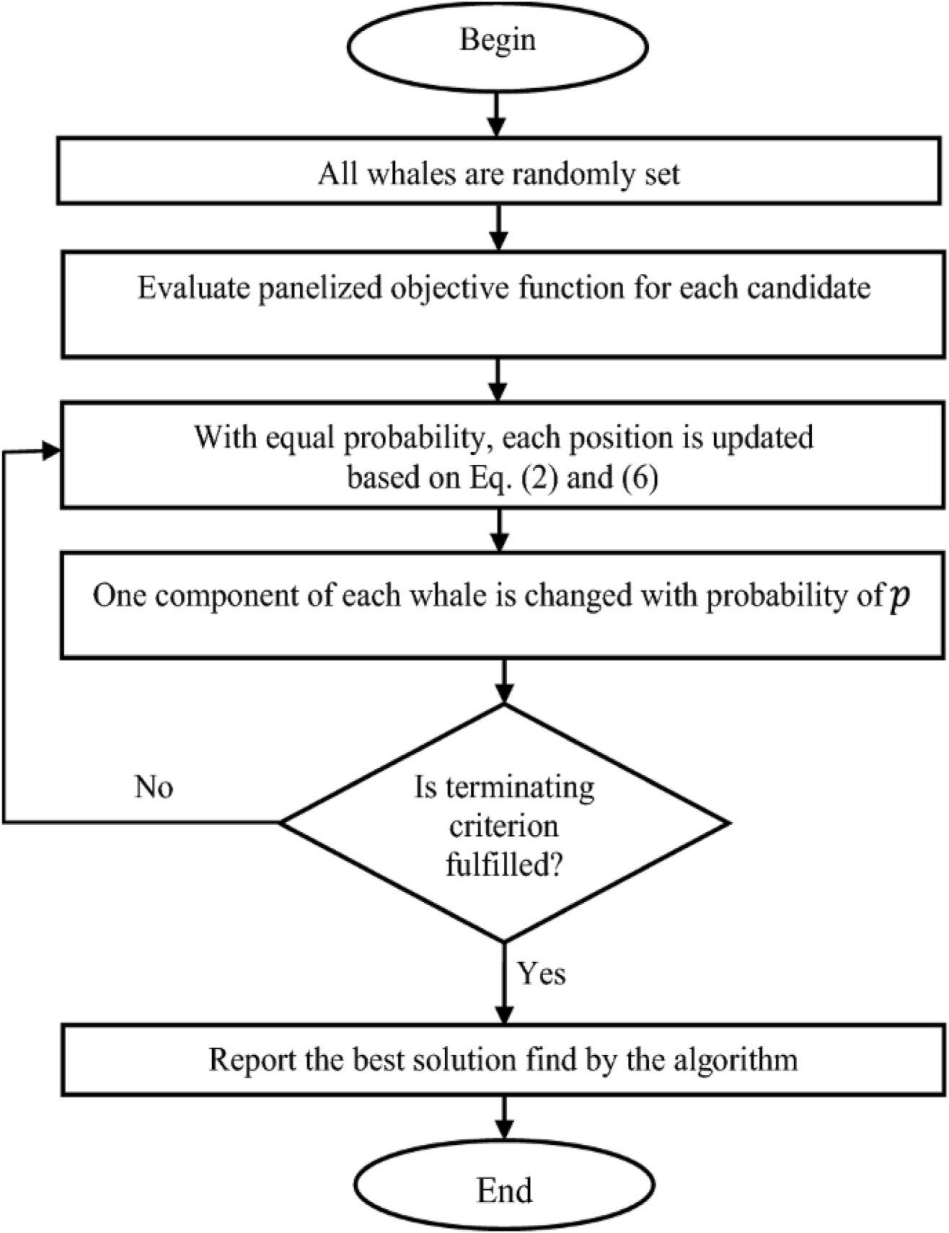
Flowchart of the whale optimisation algorithm (WOA)

The WOA was developed by Mirjalili and Lewis and is a new optimisation algorithm. It is based on a mathematical model developed by humpback whales who employ the ‘spiral bubble net’ approach to emulate the three behaviours of prey in the immediate vicinity, random search and hunting behaviour for prey. Humpback whales hunt by blowing distinctive bubbles in a ‘9’ or ‘O’ pattern while constantly approaching their prey [34]. Many academics throughout the world have been following the WOA's progress since its inception due to its impressive results. Many useful research discoveries have been obtained as a result of studying it. Year after year, there has been a huge growth in the number of published research articles on WOA and its advancement and application. This is generally the case when the correction of Gamma value utilised exceeds 1; the image's highlights have been compressed, while the shadows have been extended. Expanding highlights and compressing shadows are both effects of correction of Gamma when the value is less than 1. Lower grayscale portions of the image are stretched, whereas higher grayscale areas are compressed when gamma is less than 1. High grayscale portions of the image are squeezed and stretched when gamma is larger than 1 [35, 36]. Figure 3 offers the steps of the flowchart for WOA.

### 3.3 Classification

On the COVID lung CT images dataset, the performance of both SVM and DL architectures was investigated by first training the SVM model with the retrieved features and then the DL model by applying transfer learning [37].

#### 3.3.1 Support vector machine

Supervised learning approaches that use linear classifiers were implemented, such as SVM. In some high‐dimensional space, the input data is linearly transferred to non‐linearly separated data, resulting in outstanding classification performance. The input labelled data is analysed and the attributes are observed for the classification of subsequent unlabelled data. Support vectors are those positions closest to the marginal line that maximise the marginal distance (also known as the functional margin) between two or more intra classes. Different kernels, such as linear and (RBF) radial basis functions, are used to divide classes. A linear kernel is used as the best hyperplane between the three support vectors to distinguish the two classes in SVM represented in a 2D input space. The computational complexity of SVMs, like that of neural networks, is independent of the input space's dimensionality [38, 39]. However, the learnt function in SVM is frequently difficult to comprehend. Multiclass SVM classification has proven extensively applicable, despite the fact that SVM was originally designed to only work with two classes. To make a reliable forecast, several 2 class SVMs can be used, either by applying one versus one or one versus all. Class of winning is then described by the maximum votes or highest output function [40, 41].

#### 3.3.2 Deep learning

**FIGURE 4:**
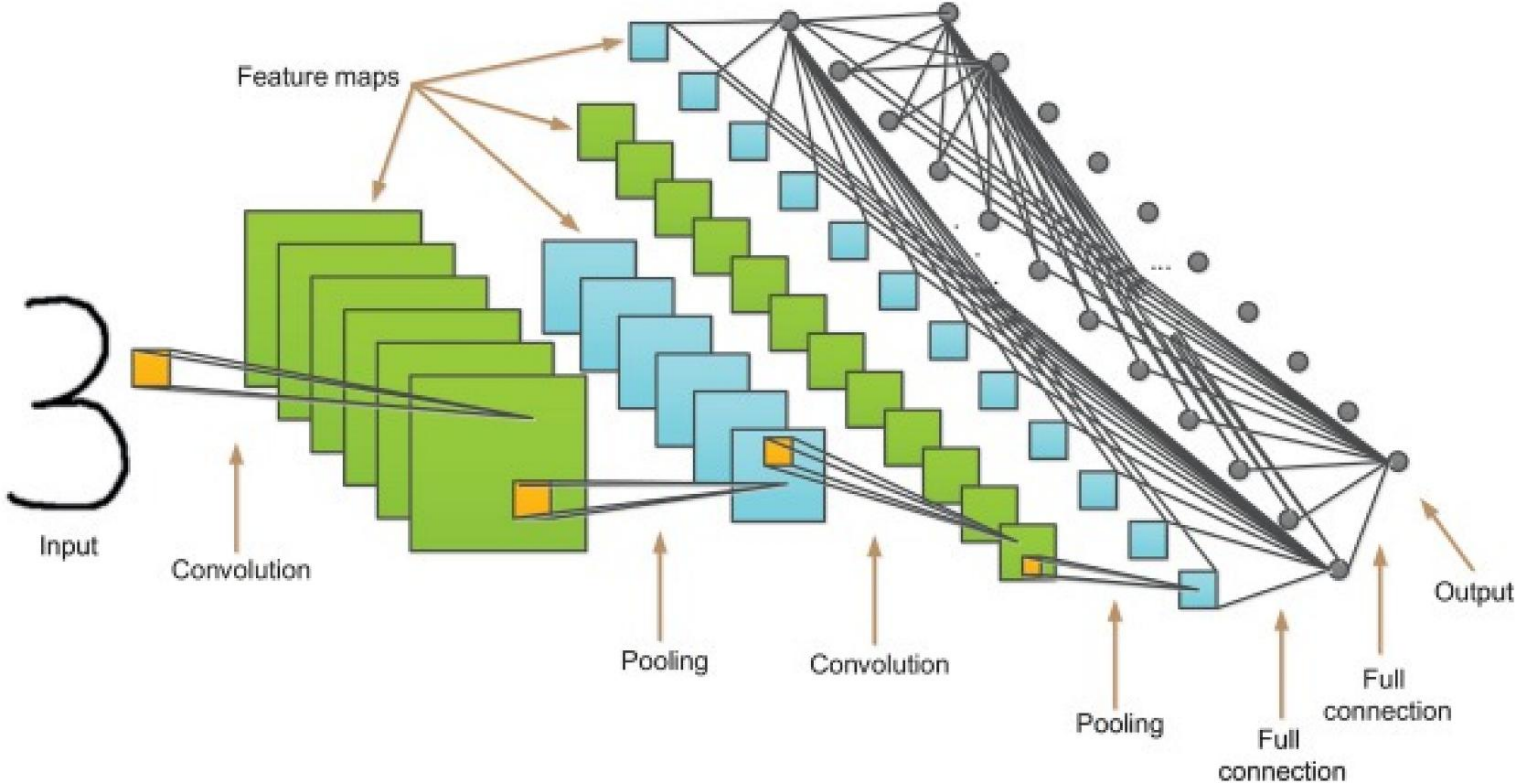
Deep convolution neural network

Deep Convolution Neural Network are being part of automated end‐to‐end learning for illness identification in a variety of fields [12]. The DL refer to a class of techniques for ML where many layers of processing for stages of information are sequenced in a nodes hierarchy to be utilised for analysis, pattern classification and unsupervised feature learning. In the deep neural network, nodes, also known as neurons, are mathematical functions that accept numerical inputs and as edges of incoming and an outgoing edge in the form of numerical output values. All neurons in the hidden layer have activation function and weight connections, adding layers means more weights and interconnections within and between the layers. To ensure the preservation of higher level features in deeper layers, DL computes observational data hierarchically. They are also classified according to whether they are from the surface or low‐level layers . The deep architecture is a term used to describe such a feature hierarchy. An image's observational data can be represented as an array of pixels. For feature learning, there are two ways to train a DL network: Transfer learning and training from scratch. Due to the deep architecture design, training a DL from ‘scratch’ is often a time‐consuming and hard task. When it comes to applying what you have learnt to new situations, this is called ‘transfer learning’ [42, 43]. This method has proven to be effective in other computer vision domain challenges and can change to meet the needs of the issue at hand. The most common architectures of transfer learning are as follows: Visual Geometry Group Net, AlexNet, GoogleNet and ResNet among others. Learning with AlexNet is now more beneficial for improving feature representations in most circumstances and decreasing the complexity of architecture [44]. For this reason, this network is employed in this study. The variations or local receptive fields in the architecture of AlexNet are commonly placed convolution layers followed by one or more fully connected layers. Layers of normalising and pooling can be placed immediately after the convolutionary ones, and traditionally, the Rectifying linear unit (ReLU) is applied to activate or initiate all layers. Each neuron's output is transformed by the ReLU and the output is then mapped to the highest possible value or zero if the value is negative [45]. It uses adaptive learning to learn the parameters of rectifiers, resulting in a significant improvement in accuracy at a minimal cost [46, 47]. Figure 4 offers deep convolution neural network.

## 4 RESULTS AND DISCUSSION

MATLAB 2018b runs on a 3.5 GHz CPU to replicate the experiments, and macOS Mojave 10.14.1 has 16 GB of RAM. In order to improve one's performance, several image processing and AI frameworks are used.

Whale Optimisation Algorithm employs a total of 10 search agents and allows for a maximum of 50 iterations. To assess the image quality, the grey value variance is utilised. Variance increases the dispersion of the greyscale histogram.

**FIGURE 5:**
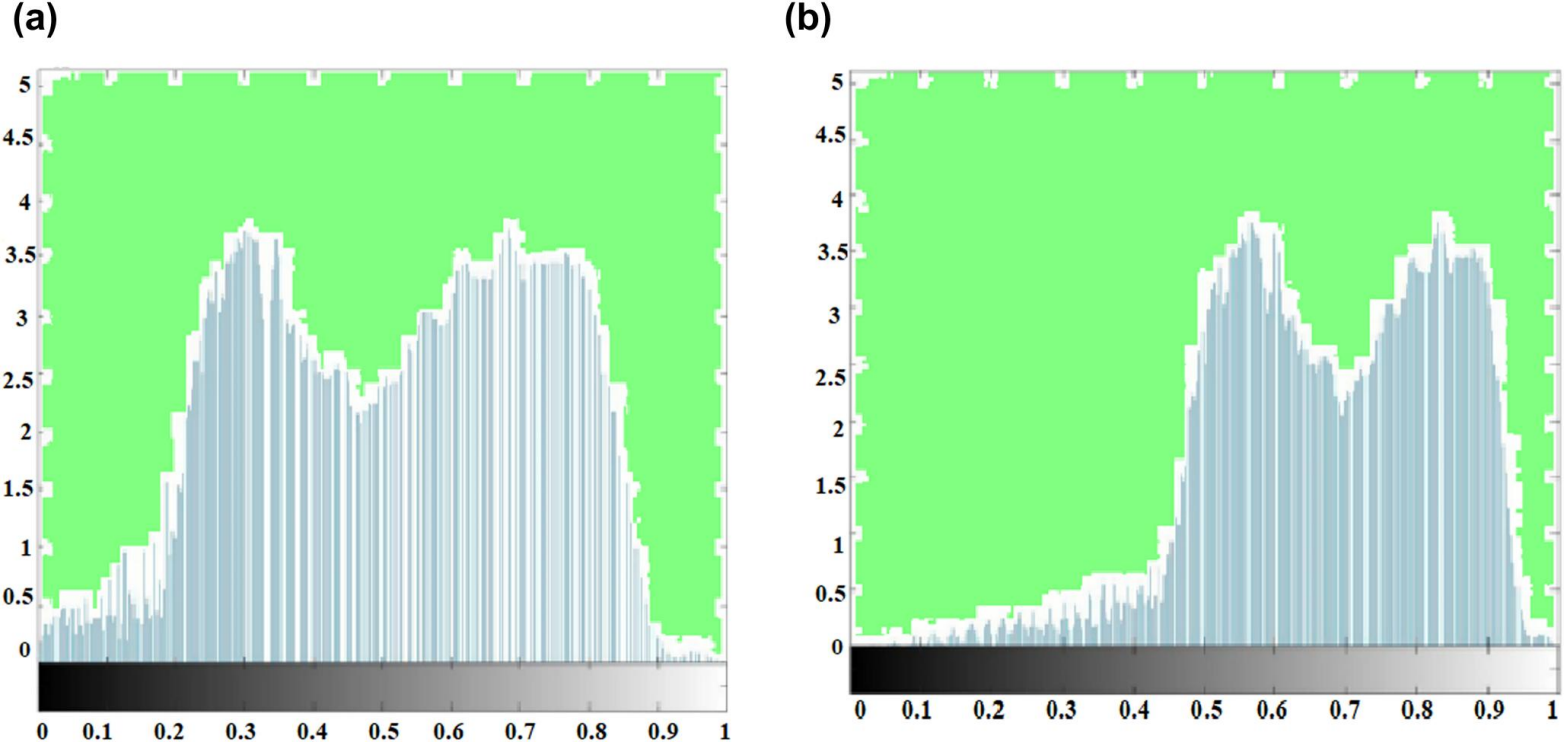
(a) Histogram of original image (b) Optimised Histogram of image

**FIGURE 6:**
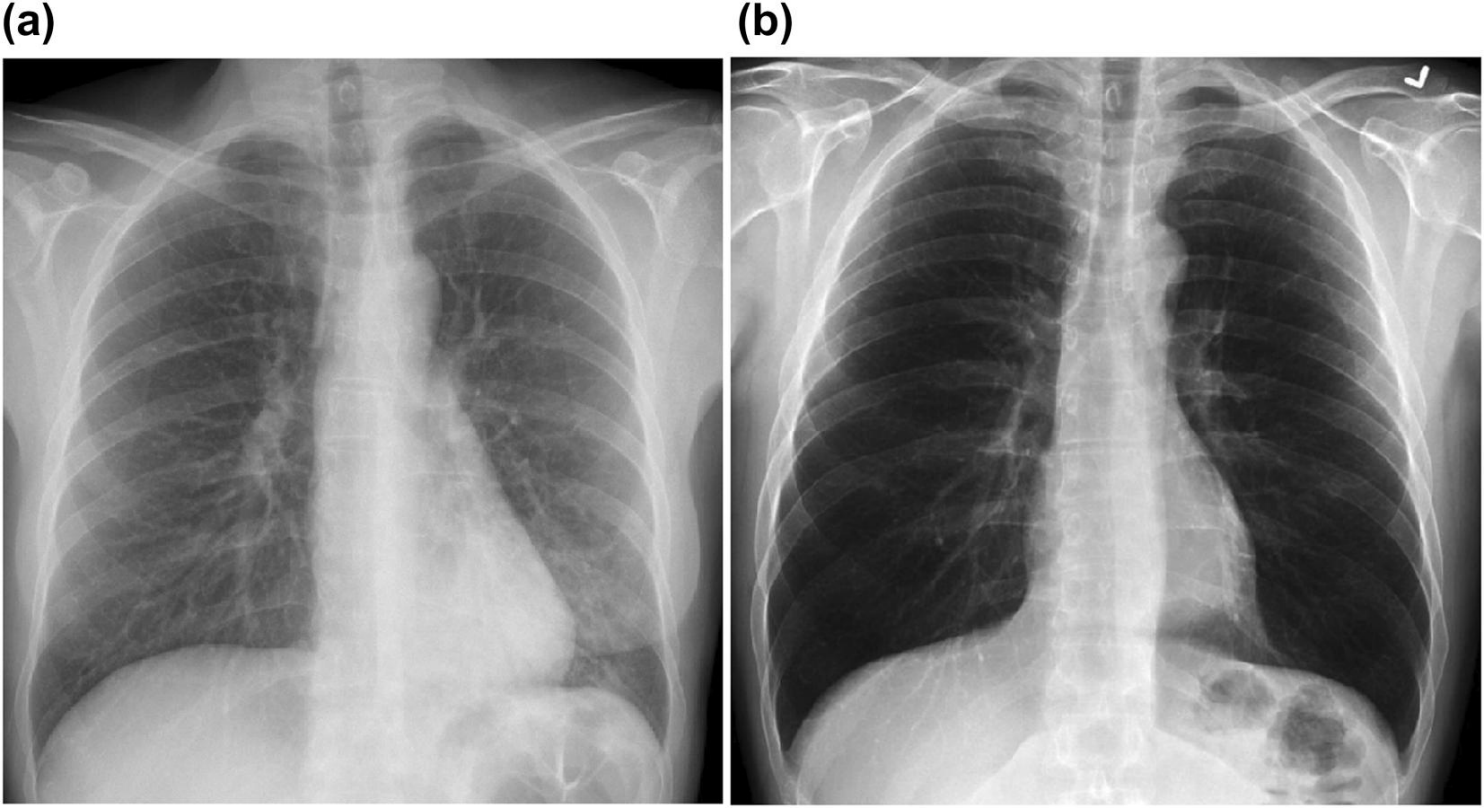
(a) Original image (b) Optimised image

**FIGURE 7:**
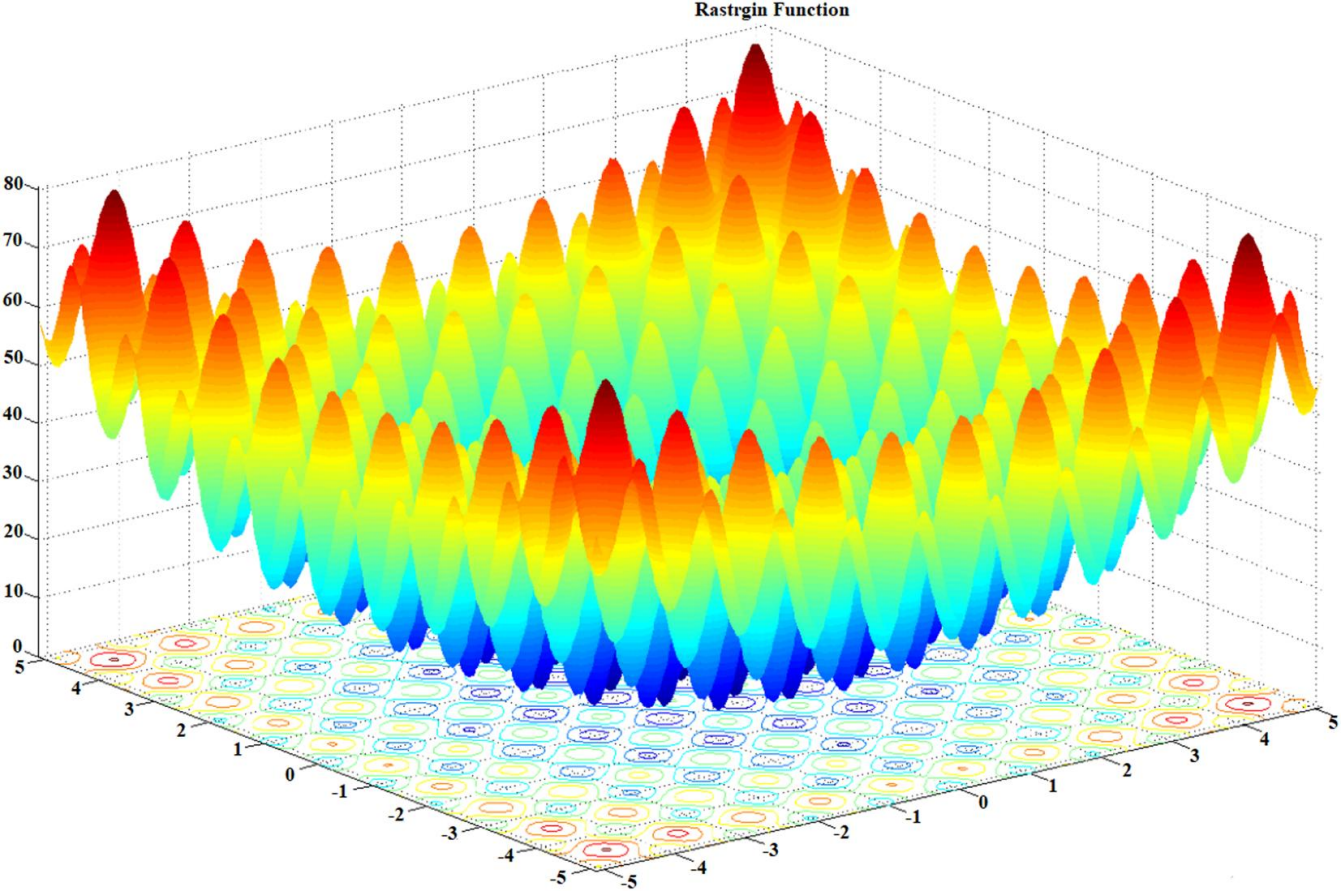
Function of Rastrigin for two variables

Figure 5 shows the gradation histogram of the original image. The gradation histogram is presented in Figure 6 using the value of WOA to optimise. The gradation histogram, as illustrated in Figures 6 and 7, has a double peak. The overall double peak is relocated towards the middle of the gradation histogram after optimisation, as seen in Figures 6 and 7. The grey values in Figure 6 is more diffused following WOA optimisation, particularly in the region of [0.2, 0].

**FIGURE 8:**
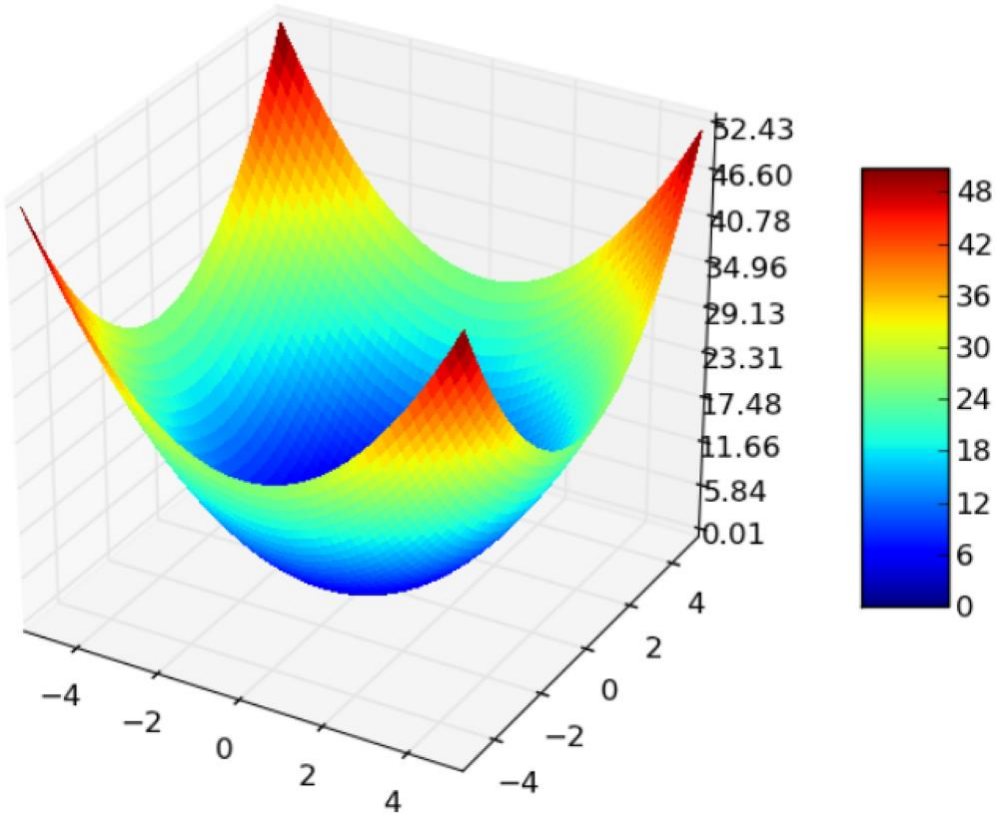
Function of Sphere for two variables

The original image is shown in Figure 5. The value of an image optimised with WOA is shown in Figure 6. The bronchus in the lungs cannot be seen in detail in Figure 5 due to the image's haziness. After improving the value, Figure 6 can better observe the features of the lung bronchus. Figure 8 shows the function of sphere for two variables. Table 1 shows the mean, median and mode values.

**TABLE 1:** Mean, median and mode values of lung computed tomography (CT) image

Image	Mean	Median	Mode	Variance
Original image	5.77	5.24	4.09	0.062
Optimised image	4.22	4.01	3.44	0.074

**FIGURE 9:**
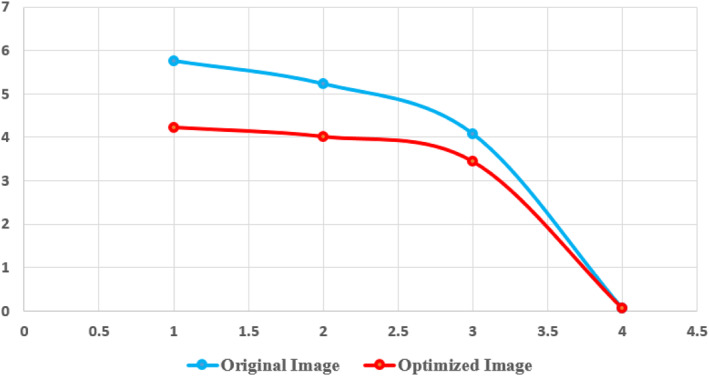
Mean, mode, median and variance

Some of the misclassifications in both models occurred because of samples exhibiting early signs of COVID illness. First, the model of SVM was trained and fine‐tuned. Mapped training data used the dot or linear kernel space utilising sequential minimisation in order to separate the hyperplane. Many distributions went through the same process. In all situations, the total time spent on the CPU for training and validation was around 37 s. Figure 9 shows the mean, mode, median and variance for original and optimised images.

**FIGURE 10:**
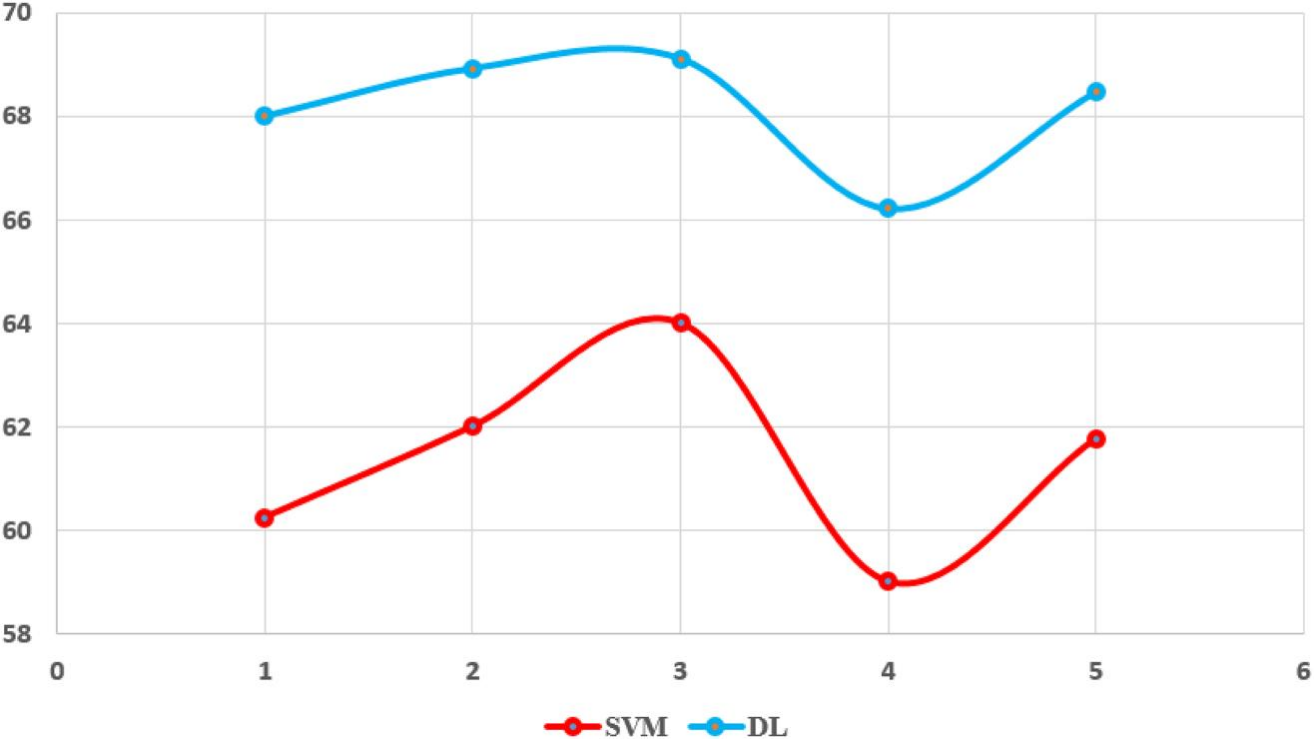
Performance of the support vector machine (SVM) and deep‐learning (DL) without whale optimisation algorithm (WOA)

The training pf SVM without feature selection algorithm achieved an accuracy rate of 92.5%, specificity, sensitivity and precision of 92.3%, and F‐Measure. While DL achieved an accuracy rate of 92.5%, specificity, sensitivity, precision of 92.3%, and F‐Measure as shown in tables and figures. From the results we concluded that DL is best from SVM. Figure 10 shows the performance of SVM and DL without WOA.

**FIGURE 11:**
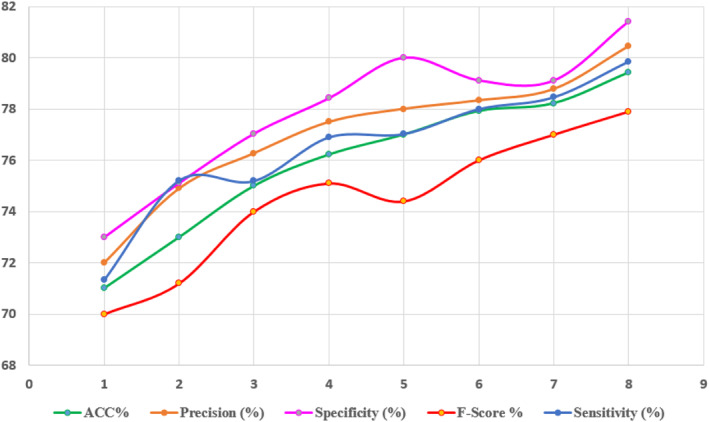
Performance of the support vector machine (SVM) on variant and number of symptoms

The most challenging part of running the SVM model is extracting the features. However, the training and validation processes were both relatively quick. Overall, SVM with WOA achieved an accuracy rate of 92.5%, specificity , sensitivity and precision of 92.3%, and F‐Measure. While DL with WOA feature selection achieved an accuracy rate of 92.5%, specificity , sensitivity and precision of 92.3%, and F‐Measure. Figure 11 shows the performance of SVM on variant and number of symptoms.

**FIGURE 12:**
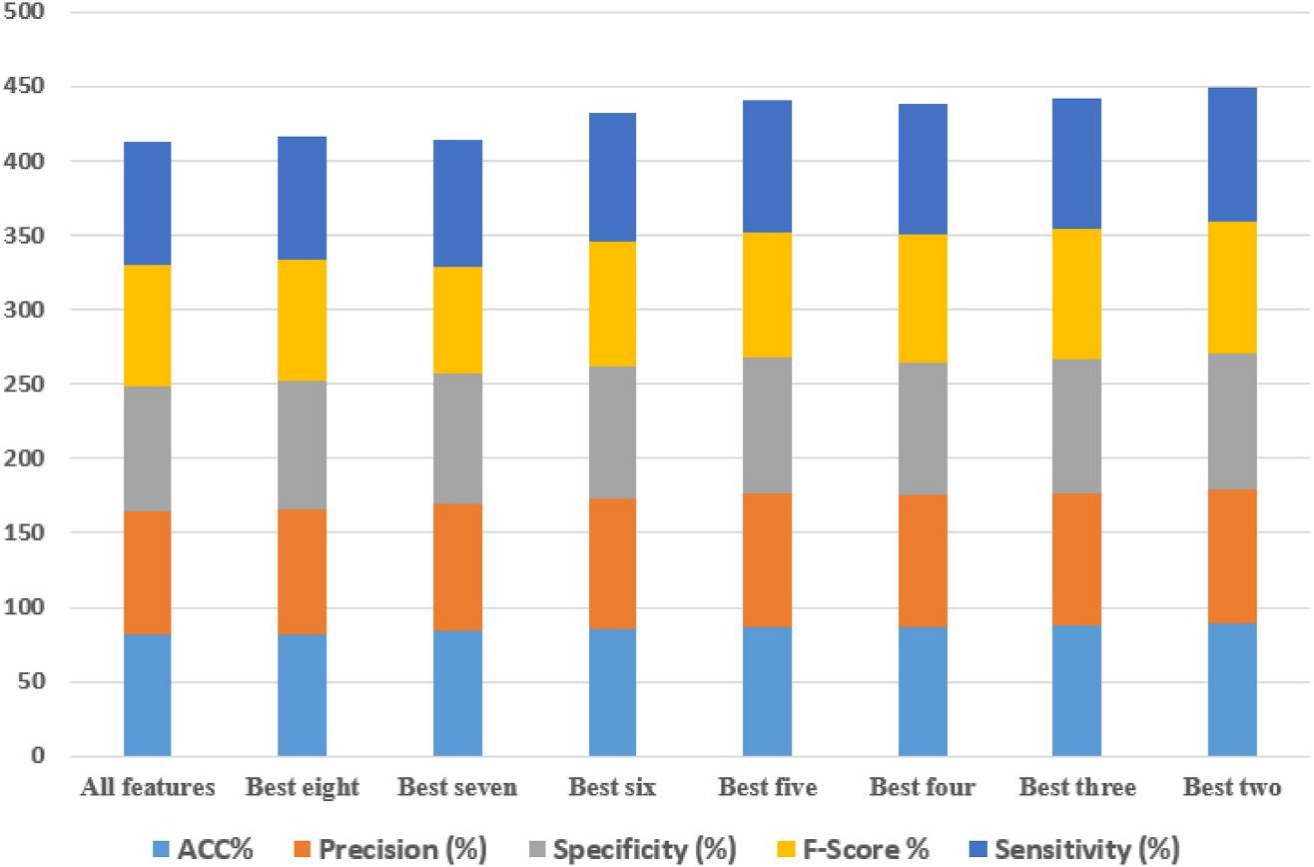
Performance of Deep‐Learning (DL) on variant and number of symptoms

K‐fold cross‐validation was used to assess the quality and quantity of nine features in SVM and DL on different distributions. First, the target's correlation with all features was calculated. As a result, the features were sorted by the strength of their link: the higher a feature's correlation weight, the better its quality as a discriminant for increased performance. It was then utilised to develop and evaluate the classifier using the first subset of all scraped features, and the feature with the lowest ranking is eliminated in each successive round, until just the two most highly rated traits remained at the end of the process. Tables 2, 3, 4 summarise the findings. Finally, Figure 12 shows the performance of DL on variant and number of symptoms.

**TABLE 2:** Performance of the support vector machine (SVM) and deep‐learning (DL) without whale optimisation algorithm (WOA)

Algorithm	ACC%	Precision (%)	Specificity (%)	F‐score %	Sensitivity (%)
SVM	60.24	62	64	59	61.77
DL	68	68.92	69.11	66.22	68.47

Abbreviation: ACC, accuracy.

**TABLE 3:** Performance of the support vector machine (SVM) on variant and number of symptoms

Subset of features	ACC%	Precision (%)	Specificity (%)	F‐score %	Sensitivity (%)
All features	71.01	72	73	70	71.34
Best eight	73	74.9	75.11	71.2	75.21
Best seven	75	76.27	77.04	74	75.19
Best six	76.22	77.49	78.42	75.1	76.89
Best five	77	78	80	74.4	77.02
Best four	77.92	78.33	79.12	76	77.99
Best three	78.22	78.77	79.11	77	78.45
Best two	79.42	80.44	81.41	77.9	79.84

Abbreviation: ACC, accuracy.

**TABLE 4:** Performance of deep‐learning (DL) on variant and number of symptoms

Subset of features	ACC%	Precision (%)	Specificity (%)	F‐score %	Sensitivity (%)
All features	82.11	83	84	81	82.91
Best eight	82.22	84.11	85.61	81.33	83.44
Best seven	84	85.02	87.77	72.11	84.99
Best six	85.77	87.49	88.42	84.12	86.57
Best five	86.19	90	91.22	84.42	88.47
Best four	87.24	88.52	89.11	86	87.99
Best three	88.12	88.77	89.72	87.32	88.34
Best two	89.22	90.44	91.04	88.91	89.84

Abbreviation: ACC, accuracy.

## 5 CONCLUSION

In the conclusion section of this paper, ML models, SVM and DL, have been offered in the investigation of COVID disease. And it is observed that DL outperforms SVM using WOA and without using WOA feature selection technique. Also, it has been determined that least features promote improvement in the accuracy of models (SVM and DL). From the findings, we observe the performance of two popular models. We offer an integrated framework of IoT that contains AI, image processing, and WOA feature selection. According to the findings, image processing‐based COVID illness detection may benefit from either of these two approaches (SVM and DL), which are within the constraints of computational power, training data and architectural complexity. The image with WOA‐optimised gamma values has stronger contrast and can offer more features of the lung bronchus.

## CONFLICT OF INTEREST

Authors confirm that there is no conflict of interest related to this work.

## Data Availability

Chest CT images and clinical metadata and codes are deposited into the China National Centre for Bio‐information at the website (http://ncov‐ai.big.ac.cn/download?lang%26equals;en).
